# Supplementary material for: WordCommentsAnalyzer: A windows software tool for qualitative research
Source: F1000Res. 2018 Sep 4;7:536. Originally published 2018 May 3. [Version 2] doi: 10.12688/f1000research.14819.2 (PMC6137411; doi:10.12688/f1000research.14819.2)
Supplement: Supplementary file 3 [file f1000research-7-17614-s0002.tgz › b2a79496-4ada-47c4-9682-a99ce867836d.zip!/Eight Iranian Health Journals/Salmand_Iranian Journal of Ageing _17.docx]

© Salmand_Iranian Journal of Ageing . This work is licensed under CC BY-NC 4.0 (https://creativecommons.org/licenses/by-nc/4.0/).

Volume & Issue: دوره 9 - شماره 1

Title: بررسی ارتباط حمایت اجتماعی و احساس رضایتمندی از زندگی در میان سالمندان ساکن آسایشگاه‌های شهر تهران

http://salmandj.uswr.ac.ir/article-1-552-fa.html

اهداف: هدف از انجام پژوهش حاضر، بررسی ارتباط حمایت اجتماعی و رضایت مندی از زندگی در سالمندان ساکن آسایشگا ههای شهر تهران بود. مواد و روش‌ها: مطالعه حاضر از نوع مقطعی‌همبستگی و جامعه آماری این تحقیق شامل تمامی سالمندان ساکن در آسایشگاه‌های شهر تهران است که از میان آنان، 150 نفر (105 نفر مرد و 45 نفر زن) سالمند ساکن در آسایشگاه‌های مناطق 3، 10 و 20 شهر تهران به‌صورت خوشه‌ای و تصادفی ساده، به‌عنوان نمونه انتخاب شدند. ابزار پژوهش شامل پرسش‌نامه حمایت اجتماعی واکس (SS-A) و پرسش‌نامه رضایتمندی از زندگی داینر بود. برای تجزیه و تحلیل داده‌ها از نرم‌افزار SPSS17 و آمار توصیفی نظیر میانگین، انحراف معیار و آمار استنباطی نظیر آزمون همبستگی پیرسون، تی مستقل، رگرسیون دومتغیره خطی به روش ورود و آزمون کلوموگروف‌اسمیرنوف جهت بررسی پارامتری نبودن داده‌ها مورد استفاده قرار گرفت. یافته‌ها: نتایج این پژوهش نشان داد که رابطه معناداری بین حمایت اجتماعی و رضایت مندی از زندگی در سالمندان ساکن آسایشگاه‌ها وجود دارد (035/P=0) ولی تفاوت معناداری در حمایت اجتماعی (082/P=0) و رضایتمندی از زندگی (061/P=0) بین سالمندان زن و مرد یافت نشد. نتایج تحلیل رگرسیون حاکی از این بود که متغیر حمایت اجتماعی در سالمندان ساکن در آسایشگاه‌ها دارای رابطه خطی با متغیر رضایتمندی از زندگی است و می‌تواند تغییرات مربوط به آن را پیش‌بینی نماید. نتیجه‌گیری: براساس نتایج تحقیق حاضر، دریافت حمایت اجتماعی از طرف خانواده، دوستان و آشنایان می‌تواند میزان رضایتمندی از زندگی در میان سالمندان ساکن آسایشگاه را ارتقا بخشد.

Title: بررسی رابطه جهت‌گیری مذهبی و کیفیت زندگی در سالمندان مرد ساکن در خانه و ساکن در خانه سالمندان شهرستان تبریز

http://salmandj.uswr.ac.ir/article-1-515-fa.html

اهداف: هدف از مطالعه حاضر، بررسی رابطه بین جهت‌گیری مذهبی و کیفیت زندگی در سالمندان ساکن در خانه و ساکن در خانه سالمندان شهرستان تبریز بود. مواد و روش‌ها: در این مطالعه توصیفی‌مقطعی، 56 مرد سالمند (20 نفر ساکن خانه سالمندان و 36 نفر ساکن در خانه) به‌صورت تصادفی و با روش نمونه‌گیری خوشه‌ای انتخاب شدند. سالمندان پرسش‌نامه‌های کیفیت زندگی جان و ویر (2000) (SF-36) و جهت‌گیری مذهبی آلپورت و راس (1967) را تکمیل نمودند. داده‌های به‌دست‌آمده به‌وسیله ضریب همبستگی پیرسون و آزمون تی برای گروه‌های مستقل مورد تجزیه‌وتحلیل قرارگرفت.  یافته‌ها: یافته‌ها نشان داد که در مردان سالمند ساکن در خانه جهت‌گیری مذهبی درونی به‌طرز مثبت و معنی‌داری با سلامت روانی، سلامت عمومی، عملکرد اجتماعی، عملکرد جسمانی و شادکامی (01/P<0) رابطه دارد و جهت‌گیری مذهبی برونی به‌طرز منفی و معنی داری با درد بدنی (01/P<0) دارای رابطه است. در مردان ساکن در خانه سالمندان نیز جهت گیری مذهبی درونی به‌طرز مثبت و معنی‌داری با سلامت روانی، سلامت عمومی، عملکرد اجتماعی، عملکرد جسمانی و شادکامی (01/P<0) رابطه داشته و جهت‌گیری مذهبی برونی به ‌طرز منفی و معنی‌داری با ایفای نقش عاطفی (05/P<0) رابطه دارد. یافته‌ها همچنین حاکی از این بود که درمجموع سالمندان ساکن خانه در مقایسه با هم‌نوعان خود در خانه سالمندان از کیفیت زندگی بهتری برخوردار هستند (01/P<0). نتیجه‌گیری: نتایج تحقیق حاضر حاکی از همبستگی مثبت میان جهت‌گیری مذهبی با برخی ابعاد کیفیت زندگی و همچنین کیفیت زندگی بهتر سالمندان ساکن خانه در مقایسه با ساکنان خانه سالمندان بود، اما در این حوزه داده‌های بیشتری برای رسیدن به روشنگری بیشتر و یافتن دلایل آن موردنیاز است.

Title: اثربخشی هنردرمانی مبتنی بر ذهن‌آگاهی بر افسردگی، اضطراب، استرس و کیفیت زندگی زنان یائسه

http://salmandj.uswr.ac.ir/article-1-589-fa.html

اهداف: پژوهش حاضر به‌منظور بررسی اثربخشی هنردرمانی مبتنی‌بر ذهن آگاهی به‌عنوان مداخله گروهی جدید در روان‌درمانی بر کاهش افسردگی، اضطراب، استرس و نیز افزایش کیفیت زندگی زنان یائسه انجام شده است. مواد و روش‌ها: 17 نفر از زنان یائسه 47 تا 60 سال، در یک طرح تجربی پیش‌آزمون-پس‌آزمون با گروه گواه، از طریق نمونه‌گیری داوطلبانه انتخاب و به‌طور تصادفی به دو گروه آزمایشی و گواه به‌ترتیب به 9 و 8 نفر تقسیم شدند و به نسخه کوتاه مقیاس اضطراب، افسردگی، استرس و نیز پرسش‌نامه کیفیت زندگی سازمان بهداشت جهانی پاسخ دادند. گروه آزمایش، 10 هفته هنردرمانی مبتنی بر ذهن‌آگاهی را دریافت کردند و یک ماه پس از مداخله، هر دو گروه دوباره با همان ابزارها ارزیابی و داده‌ها با تحلیل کواریانس چندمتغیره تحلیل شدند.  یافته‌ها: اجرای هنر درمانی مبتنی بر ذهن‌آگاهی در مورد گروه آزمایش موجب کاهش افسردگی و استرس و افزایش کیفیت زندگی آن‌ها شد؛ ولی اثر معناداری روی اضطراب نداشت.  نتیجه‌گیری: هنردرمانی مبتنی بر ذهن‌آگاهی به‌عنوان یک روش جدید که ترکیبی از درمان‌های روان‌شناختی و هنردرمانی است، در بهبود افسردگی و کیفیت زندگی جمعیت زنان یائسه مؤثر است. با توجه به سودمندی این روش، استفاده گسترده از این روش در چنین جمعی تهایی پیشنهاد می‌گردد.

Title: تأثیر یک برنامه مداخله‌ای تمرینی بر عوامل روان‌شناختی مرتبط با افتادن در میان مردان سالمند شهر اراک

http://salmandj.uswr.ac.ir/article-1-498-fa.html

اهداف: افتادن و عوامل فیزیولوژکیی و روا نشناختی مرتبط با آن، از مشکلات اصلی زندگی سالمندان است. هدف این تحقیق، برّرسی تأثیر یک برنامه مداخل های تمرینی بر عوامل روا نشناختی مرتبط با افُتادن در میان مردان سالمند ساکن شهر اراک می باشد.  مواد و روش‌ها: در این تحقیق نیمه تجربی که بر روی مردان سالمند ساکن شهر اراک انجام شد، 27 آزمودنی به طور تصادفی به دو گروه کنترل (میانگین سنّی 6/65±70/21 سال) و گروه آزمایش (میانگین سنّی 4/38±66/07 سال) اختصاص یافتند. گروه آزمایش در یک برنامه مداخله تمرینی 12هفته‌ای (سه جلسه در هفته و هر جلسه 90 دقیقه) شرکت کردند.  یافته‌ها: یافته‌های تحقیق نشان داد که برنامه تمرینی اعمال شده باعث بهبود عوامل رو انشناختی مرتبط با اُفتادن (خودکارآمدی اُفتادن، ترس از افتادن و اعتماد به تعادل ویژه فعّالیّت‌ها، خود کارآمدی تعادل) در سالمندان گروه آزمایش گردید. نتایج گروه کنترل که هیچ‌گونه برنامه تمرینی منظّمی را دنبال نکردند و نیز عدم تغییر در عوامل روان‌شناختی مرتبط با افتادن را نشان داد.  نتیجه‌گیری: براساس یافته‌های این تحقیق، برنامه مداخله‌ای تمرینی منظّم می‌تواند با ارتقای سطح آمادگی جسمانی و حرکتی مردان سالمند باعث کاهش ترس از افُتادن و افزایش اعتماد به تعادل در حین انجام فعّالیّت‌های زندگی روزانه‌شان شود. این بهبودها می‌توانند به ارتقای سلامتی جسمانی و روانی و بهبود کیفیّت زندگی سالمندان ساکن در جامعه و نهایتاً سالمندی موفّقیّت‌آمیز آن‌ها کمک کند.

Title: بررسی ارتباط کیفیت زندگی مرتبط با صوت (VHI) در افراد مبتلا به بیماری پارکینسون فارسی‌زبان

http://salmandj.uswr.ac.ir/article-1-573-fa.html

اهداف: در سال‌های اخیر، ارزیابی اختلالات صوت از روش‌های سنتی به رویکردهای جامع‌تری حرکت کرده که دربرگیرنده کیفیت زندگی بوده است. کیفیت زندگی مرتبط با صوت (VHI) پرکاربردترین ابزار «خودارزیابی » در بیماران دچار «معلولیت صوت» است. در بیماری پارکینسون صوت سریع‌تر و بیش‌از سایر زیرسیستم‌های گفتار تحت‌تأثیر قرار می گیرد. هدف این پژوهش، بررسی معلولیت ناشی از اختلال صوت متعاقب پارکینسون در میان فارسی‌زبانان است. مواد و روش‌ها: در این مطالعه توصیفی‌تحلیلی، ۵۰ مُراجع مبتلا به پارکینسون (۳۵ مرد ۱۵ زن) از بیمارستان حضرت رسول‌اکرم(ص) دانشگاه تهران به روش نمونه‌گیری دردسترس انتخاب و اطلاعات دموگرافیک آن‌ها ثبت شد. با تکمیل نسخه فارسی VHI از طریق بیماران، به کمک نرم‌افزار SPSS نسخه 18 نمره VHI-T و سه حوزه عملکردی، فیزیکی و عاطفی اختلال صوت در کل بیماران و به تفکیکِ جنس به‌دست آمد. یافته‌ها: ۸۳درصد بیماران، معلولیت ناشی از اختلال صوت را گزارش کردند و بین VHI-T و زیرآزمون‌هایش در دو جنس، تفاوتی دیده نشد. VHI-T و حوزه‌های عملکردی و عاطفی با سن رابطه مثبت داشتند. رابطه VHI-T و بخش عملکردی نیز با دیرش بیماری مثبت بوده و در مردان VHI-T و هر سه زیرآزمونش با دیرش ارتباط مثبت داشتند. نتیجه‌گیری: اغلب فارسی‌زبانان مبتلا به پارکینسون، اختلال صوت ناشی از بیماری را بر کاهش کیفیت زندگی خود مؤثر می‌دانند. افزایش سن و دیرش بیماری، بر اختلال صوت می‌افزاید و از کیفیت زندگی خواهد کاست؛ به‌خصوص بیماران از نظر عملکردی، معلولیت بیشتری را نسبت‌به اختلال صوت خود احساس می کنند. به نظر می‌رسد که مردان با افزایش زمان بیماری، بیش‌از زنان احساس معلولیت می‌کنند.

Title: فراتحلیل اثربخشی مداخلات روان‌درمانی بر میزان افسردگی سالمندان ایران 89-1379

http://salmandj.uswr.ac.ir/article-1-509-fa.html

اهداف: در سال‌های اخیر درمانگران در زمینه افسردگی سالمندان، به‌دنبال سنجش اعتبار و کارآیی مداخلات روان‌شناختی و مشاوره‌ای بوده‌اند. در این خصوص، فراتحلیل با یکپارچه‌کردن نتایج حاصل از تحقیقات مختلف، میزان اندازه اثر و اثربخشی مداخلات روان‌شناختی را مشخص می‌نماید. مواد و روش‌ها: پژوهش حاضر نیز بر آن است تا با استفاده از الگوی پژوهشی فراتحلیل، میزان اثربخشی مداخلات روان‌شناختی را بر افسردگی سالمندان، موردپژوهش قرار دهد. با توجه به ناهمخوانی‌هایی که در نتایج تحقیقات مربوط به اثربخشی مداخلات روان‌شناختی بر افسردگی سالمندان وجود دارد، به‌نظر می‌رسد که انجام یک فراتحلیل به روشن‌ساختن مقدار واقعی تأثیر مداخلات روان‌شناختی بر افسردگی سالمندان کمک خواهد کرد و نیز میزان اثربخشی مداخلات روان‌شناختی بر کاهش افسردگی سالمندان را نشان خواهد داد. به‌عبارتی روشن‌تر، با انجام فراتحلیل می‌توان دیدگاه کلی‌تری نسبت‌به تأثیر مداخلات روان‌شناختی در کشور به‌دست آورد. این روش به پژوهشگر امکان آزمایش فرضیه‌ای را می‌دهد که در مطالعات مختلف مطرح‌شده و موردآزمایش قرار گرفته است. یافته‌ها: بدین‌منظور از بین 20 پژوهش، 9 پژوهش که از لحاظ روش‌شناختی موردقبول بوده‌اند انتخاب شد و فراتحلیل بر روی آن انجام گرفت. بیشترین حجم اندازه اثر 0/58 مربوط به پژوهش صدهزاری و همکاران و کمترین حجم آن در این تحقیق 0/26 مربوط به پژوهش شیبانی تذرجی و همکاران است. ابزار پژوهش عبارت بود از: چک‌لیست فراتحلیل. میزان اندازه اثر نیز 0/43 است (P<0/001). نتیجه‌گیری: نتایج فراتحلیل نشان داد که میزان اندازۀ اثر مداخلات روان‌شناختی بر کاهش افسردگی سالمندان مطابق جدول تفسیر اندازه اثر کوهن، متوسط ارزیابی می‌شود.

Title: بررسی پیامدهای ناشی از سقوط در سالمندان مراجعه‌کننده به بیمارستان امام حسین شهر تهران در سال‌های 89-1388

http://salmandj.uswr.ac.ir/article-1-357-fa.html

اهداف: سقوط یکی از دلایل شایع حضور سالمندان در بخش های اورژانس می باشد. در پی سقوط نه تنها سالمند رنج و درد فراوانی را متحمل می شود، بلکه هزینه اقتصادی کلانی را نیز برای وی به همراه دارد؛ لذا تعیین میزان ترومای وارده به سالمند در پی سقوط، بسیار مهم می باشد. این مطالعه به منظور بررسی پیامدهای ناشی از سقوط و سنجش کمی شدت ترومای متعاقب سقوط در بیماران سالمند انجام شد. مواد و روش‌ها: در این مطالعه مقطعی 118 بیمار سالمندی که در پی سقوط به بیمارستان اما محسین مراجعه کرده بودند موردمطالعه قرار گرفتند. جهت گردآوری داده‌ها از چک‌لیست استفاده شد. در انتها میزان شدت ترومای وارده توسط مقیاس‌های ISS ،RTS و TRISS اندازه‌گیری شد.  یافته‌ها: نتایج نشان داد که علت سقوط در 67درصد از موارد شامل لیزخوردن و 33درصد سنکوپ بوده است. 22درصد از بیماران فوت نموده، بیش از نیمی از آنان دچار شکستگی استخوان شده و 41درصد از اجرای عملکردهای روزانه خود ناتوان شدند. میانگین ISS 8/9±12/06، میانگین 4/52±RTS 6/54 و میانگین 0/33±TRISS 0/45 بوده است. میانگین نمرات مقیاس‌های محاسبه شده ارتباط معناداری با شکستگی استخوان و مرگ بیماران داشتند و کلیه مقیاس‌ها نیز در افراد دچار مورتالیتی و موربیدیتی بالاتر بودند (P<0/05). نتیجه‌گیری: با توجه به اینکه با افزایش سن، حوادث و بیماری‌هایی که گریبان‌گیر افراد می‌شوند روبه‌افزایش است، ضرورت اجرای تدابیر به ‌موقع جهت درمان، مدیریت صحیح و پیشگیری از عواقب تروما احساس می‌شود.

Title: وضعیت به‌کارگیری اینترنت و ارتباط آن با احساس تنهایی در بازنشستگان فرهنگی

http://salmandj.uswr.ac.ir/article-1-698-fa.html

اهداف: حفظ روابط و مشارکت اجتماعی سالمندان در جامعه از عوامل پیشگوی سالمندی موفق به‌شمار می‌رود. با تغییراتی که در سال‌های اخیر در ساختار جامعه به‌وقوع پیوسته است، سالمندان زیادی در معرض خطر احساس تنهایی قرار گرفته‌اند. مطالعات بیانگر آن است که تنهایی اثرات مخربی بر سلامت سالمندان دارد؛ لذا یافتن راهکارهایی برای مقابله با این مشکل بسیارمهم است. هدف از انجام این پژوهش، تعیین وضعیت به‌کارگیری اینترنت و ارتباط آن با احساس تنهایی در بازنشستگان فرهنگی شهر بابل بوده است. مواد و روش‌ها: این مطالعه مقطعی روی 445 معلم بازنشسته سالمند که 43 نفر از آنان از کاربران اینترنت بوده‌اند، انجام شده است. برای جمع‌آوری اطلاعات از پرسش‌نامه‌هایی مشتمل بر 3 بخش استفاده شده است که ویژگی‌های جمعیت‌شناختی 15 سؤال در زمینه احساس تنهایی و 5 سؤال در زمینه تعیین میزان افسردگی را دربرمی‌گیرند. نتایج تحلیل فاکتورها نشان داد که ابزار احساس تنهایی از 3 زیر فاکتور احساس تنهایی اجتماعی، خانوادگی و رمانتیک تشکیل شده است. علاوه‌بر سؤالات فوق، یک بخش دیگر نیز برای بررسی کاربران مورد استفاده قرار گرفت که مشتمل بر سؤالاتی در زمینه میزان، تاریخچه و نوع استفاده از اینترنت بود. تحلیل داده‌ها نیز با نرم افزار SPSS صورت گرفت. یافته‌ها:  37درصد از غیرکاربران در مقایسه با 18/6درصد از کاربران اینترنت، احساس تنهایی اجتماعی را گزارش کرده‌اند که تفاوت معناداری را نشان می‌دهد (002/P=0). اگرچه میزان احساس تنهایی خانوادگی در غیرکاربران به وضوح کمتر از کاربران بوده است (001/P=0). تنها زندگی کردن و بیوه یا مطلقه‌بودن نیز از عوامل پیشگویی‌کننده احساس تنهایی بوده‌اند (05/P<0). در زمینه افسردگی یافته‌ها نشان داد که میزان افسردگی در کاربران اینترنت به‌طور معناداری کمتر از غیرکاربران بوده است (002/P=0) (27/9درصد در مقایسه با 39/2درصد). نتیجه‌گیری: درمجموع نتایج این پژوهش بیانگر آن است که میزان احساس تنهایی و افسردگی در سالمندان غیرکاربر اینترنت بیشتر از کاربران می باشد؛ لذا یافتن و به‌کارگیری راهکارهایی نظیر استفاده از اینترنت برای مقابله با احساس تنهایی که توسط این قشر از جامعه احساس می‌شود، ضروری به‌نظر می‌رسد.
